# Thryroid Hormones and Hematological Indices Levels in Thyroid Disorders Patients at Moi Teaching and Referral Hospital, Western Kenya

**DOI:** 10.1155/2013/385940

**Published:** 2013-04-15

**Authors:** M. A. Iddah, B. N. Macharia, A. G. Ng'wena, A. Keter, A. V. O. Ofulla

**Affiliations:** ^1^Department of Biomedical Science and Technology, Maseno University, P.O. Box 333-40105, Maseno, Kenya; ^2^Department of Human Pathology, School of Medicine, Moi University, P.O. Box 4606-30100, Eldoret, Kenya; ^3^Department of Medical Physiology, School of Medicine, Maseno University, P.O. Box 333-40105, Maseno, Kenya; ^4^Department of Research, USAID-AMPATH Partnership, P.O. Box 4606-30100, Eldoret, Kenya

## Abstract

*Problem Statement*. Thyroid disorders are prevalent in western Kenya, but the effects of disorders on thyroid hormones and hematological indices levels have not been documented. *Study Population*. Patients treated for thyroid disorders at the MTRH between January 2008 and December 2011. *Objectives*. To determine the thyroid hormones and hematological indices levels in thyroid disorders patients at the MTRH, western Kenya. *Methodology*. A retrospective study in which patient data and stored samples of patients, who presented with thyroid pathologies, underwent thyroidectomy, and histological examinations are done. Thyroid stimulating hormone (TSH), thyroxine (T_4_), and triiodothyronine (T_3_) blood levels, white blood cells (WBCs), red blood cells (RBCs), platelet counts, and hemoglobin (Hb) levels were analyzed. *Results*. Male : female ratio was 1 : 10.9 with female representing 368 (95%). The median age was 41 (IQR: 32–48) with a range of 14–89 years. HHormonal levels for immunological thyroid disease patients were higher (*P* = 0.0232; 0.040) for TSH and (T_3_) for those aged 30–39 years, respectively. The WBCs, RBCs, HGB, and platelets in immunological thyroid disease were not statistically significant with *P* values of 0.547, 0.205, 0.291, and 0.488 respectively. *Conclusion*. The presence of anaemia due to low RBCs in thyroid disease is not significantly associated with thyroid hormone with a *P* value of 0.512.

## 1. Introduction

Thyroid hormones (THs) play an important physiological role in humans. THs may regulate human hematopoiesis in the bone marrow [1]. The association of thyroid disorders and abnormalities in hematological parameters is well known. In 1979, Fein showed that Graves' disease is associated with anemia [2]. Horton observed a decreased number of red blood cells (RBCs) in the peripheral blood (PB) of patients after thyroidectomy [3]. Hypothyroidism can cause certain forms of anemia on the one hand or hyperproliferation of immature erythroid progenitors on the other hand. The anemia is usually macrocytic hypochromic anemia of moderate severity [3]. In contrast, anemia is not frequently observed in patients with hyperthyroidism, whereas erythrocytosis is fairly common [2, 4]. It has been found that all hematological parameters return to normal when a euthyroid state is achieved [5]. As far as white blood cells and thrombocytes are concerned, a slightly depressed total leucocyte count, neutropaenia, and thrombocytopenia have been observed in hypothyroid patients [6]. Furthermore, elevated, normal, or slightly depressed total leucocyte counts have been found in hyperthyroid patients, with only a relative decrease in the number of neutrophils and a relative increase in the number of eosinophils and mononuclear cells (MNCs). Nevertheless, hyperplasia of all myeloid cell lines in hyperthyroidism and their hypoplasia in hypothyroidism were reported by Axelrod [7]. 

With regard to lymphocytes, triiodothyronine (T_3_) has been shown to be a prerequisite for normal B-cell production in the bone marrow through its regulation of pro-B-cell proliferation [8–10]. These observations confirmed the association between thyroid gland dysfunction and haematopoiesis. Previously published studies suggested that there is an essential relationship between the hypothyroid state and low levels of iron, vitamin B_12_, and folic acid in the human body [3, 11]. Furthermore, it has been postulated that the influence of THs on haematopoiesis involves an increased production of erythropoietin or haematopoietic factors by non erythroid cells [12, 13]. However, a growing number of studies have demonstrated a direct role of THs in normal human and animal erythropoiesis [1, 14–17].

## 2. Material and Methods

### 2.1. Study Site and Design

This research was carried out at Moi Teaching and Referral Hospital, Eldoret. This is a hospital that serves clients from all over North Rift, parts of western Kenya, and Nyanza province. 

This was a retrospective study in which all patients with thyroid pathologies and who underwent thyroidectomy at the MTRH between 2008 and 2011 were included.

### 2.2. Study Methods

Data on thyroid hormones and hematological indices was obtained from the medical records. Clinical data was obtained from the hospital record files for purposes of documenting the disease trends over the past four years (2008–2011). 

### 2.3. Ethical Considerations

Institution review ethics committee (IREC) approval was obtained before starting data collection. Findings were discussed with the relevant health provider. Information was provided in appropriately accessible language.

### 2.4. Thyroid Hormone Measurements

Triiodothyronine hormone (T_3_), thyroxine hormone (T_4_), and thyroid stimulating hormone (TSH) levels were measured using Enzyme Linked Immunosorbent Assay (ELISA) for quantitative determination of hormones concentration in human serum/plasma using the methods of Helenius [18]. Whole blood samples were collected through venipuncture, centrifuged at 3000 rpm, and then frozen at −20°C for storage if to be measured later.

### 2.5. Determination of Hematological Profile

Hemoglobin concentration was determined by a colorimetric method with the addition of a sample centrifugation (1,600 ×g, 5 min) before reading [19]. Erythrocytes (red blood cells) and thrombocytes were counted simultaneously in a Neubauer chamber using the modified Dacie's fluid with the addition of brilliant blue cresyl [20]. Mean corpuscular volume and mean concentration of corpuscular hemoglobin were calculated accordingly using the methods of [21]. 

The leukocyte concentration (white blood cells (WBCs)) was obtained through the counting of these cells in a Neubauer chamber using heparinized blood. Because heparin causes leukocyte destruction in ostrich blood [22], the WBC concentration was also indirectly determined by a method described previously [23]. Briefly, leucocytes and erythrocytes were counted separately along the smear up to a total of 2,000 cells; a ratio was determined, and the WBCs concentration was indirectly calculated using the red blood cells count performed as described previously. The differential count of leukocytes was made in blood smears stained with Diff-Quick.

### 2.6. Statistical Analysis

Data analysis was done using STATA version 10 SE (College Station, TX, USA). Categorical variables were summarized as frequencies (percentage), while the continuous variables were summarized as median (interquartile range). Nonparametric test of equality of medians was used to compare the medians of different groups. Wilcoxon rank-sum test was used to test whether any two groups came from populations with the same distribution. The age was categorized at the median. The comparison of the proportions was done using the test of proportions. The cutoffs for anaemia were taken as Hb < 13 g/dL for male and Hb < 12 g/dL for female patients not pregnant (http://en.wikipedia.org/wiki/Anemia, accessed on 15th August 2012 at 12:00 noon). Apparently our sample did not contain any pregnant woman. The cutoffs for low WBCs and low platelets were WBCs < 4 k/*μ*L and platelets < 150 M/*μ*L, respectively. The red blood cells (RBCs) cutoffs were RBCs < 4.5 M/*μ*L for male and RBCs < 4.2 M/*μ*L for female. These cutoffs were obtained from http://www.chemocare.com/managing/low_blood_counts.asp accessed on 15th August 2012 at 12:00 noon. The elevated levels of the hormones T_3_, T_4_, and TSH were based on the following cutoffs: T_3_ > 4.2 ng/dL, T_4_ > 1.8 ng/dL and TSH > 5.0 mIU/L.

## 3. Results

There were 388 subjects aged between 14–89 years who were eligible for analysis and were categorized as having been diagnosed with thyroid gland disorders, autoimmune thyroid disease or not.

Male : female ratio was 1 : 10.9 with female representing 368 (95%). The median age was 41 (IQR: 32–48) with a range of 14–89 years. There were 80 (28.4%, 95% confidence limits: 23.1–33.7) anemic patients (Hb < 13 g/dL if male and Hb < 12 g/dL if female) with a median Hb value of 13.2 g/dL (IQR: 11.9–14.0). The absolute WBCs count was less than 4 k/*μ*L in 13 (12.2%) of the patients with median value of 5.5 k/*μ*L (IQR: 4.2–7.3). Thrombocytopenia was seen in 5 (4.7%) patients with median platelet count of 296 × 10^3^ M/*μ*L. There were 10 (9.4%) patients and 35 (32.7%) patients with low RBC, respectively. The median RBCs were 4.6 (IQR: 4.4–4.9). There were elevated T_3_, T_4_, and TSH in 11 (10.3%), 18 (16.8%), and 9 (8.4%) patients, respectively. Hormonal levels for immunological thyroid disease patients were higher (*P* = 0.0232; 0.040). The significance is seen in thyroid stimulating hormone levels (TSH) and triiodothyronine hormone (T_3_) only for those aged 30–39 years, respectively. The WBCs, RBCs, HGB, and platelets among the immunological thyroid disease patients were WBC: 5.2 (4.1–7.0), RBC: 4.6 (4.4–4.8), HGB: 13.1 (11.6–13.9), and platelets: 292 (224–390), respectively, compared to the nonimmunological thyroid disease patients. However, the differences were not statistically significant with *P* values of 0.547, 0.205, 0.291, and 0.488, respectively. There were 9 (45%) immunological patients among the male patients. The hematological profiles as well as the age for the immunological patients were not significantly different from those of the nonimmunological thyroid disease patients.

Table 1 shows the overall distributions of age, hormones, and hematological properties which are also stratified by whether immunological thyroid disease is present or absent. The median test showed that there was only statistically significant difference in T_3_ between the two groups of patients.

Table 2 shows the relationship between thyroid disorders and hematological profiles among the male patients. There were 9(45%) immunological patients among the male patients. The hematological profiles as well as the age for the immunological patients were not significantly different from those of the non-immunological thyroid disease patients (Table 2). This may be attributable to the small number of male patients in this study. Further the autoimmune thyroid disease is rare among the male population.

**Figure 1:**
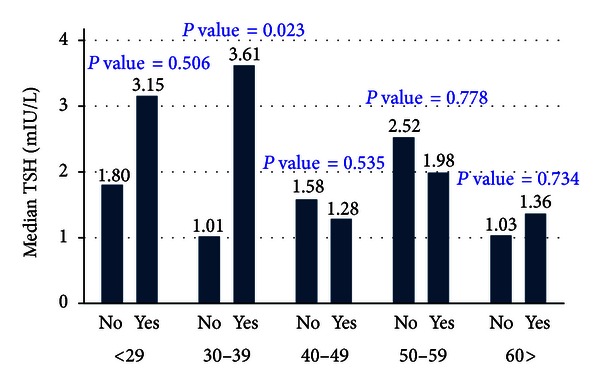
Thyroid stimulating hormone (TSH) levels among the immunological thyroid disease patients.

Figure 1 shows the thyroid stimulating hormone (TSH) levels of the subjects suffering from autoimmune thyroid disease (Yes) and those not suffering from the autoimmune thyroid disease (No) stratified by age groups. The TSH serum levels were high for those subjects suffering from the autoimmune thyroid disease across all the age groups except for those aged 40–49 and 50–59 years. However, the differences were not statistically significant at 5% level of significance except for those aged 30–39 years (*P* value = 0.023).

**Figure 2:**
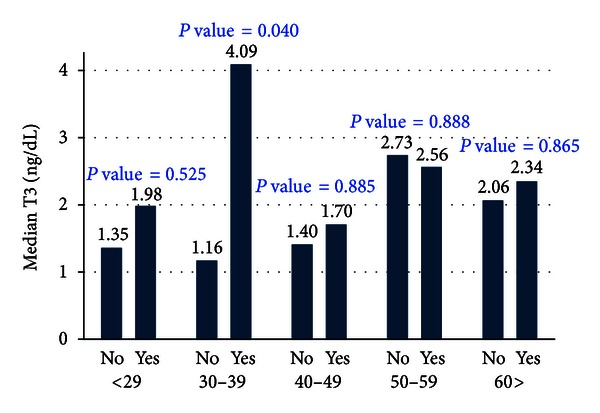
Triiodothyronine (T_3_) levels among the immunological thyroid disease patients.

The triiodothyronine (T_3_) levels were high for those subjects suffering from the autoimmune thyroid disease across all the age groups except for those aged 40–49 and 50–59 years as shown in Figure 2. However, the differences were not statistically significant at 5% level of significance except for those aged 30–39 years (*P* value = 0.040). 

**Figure 3:**
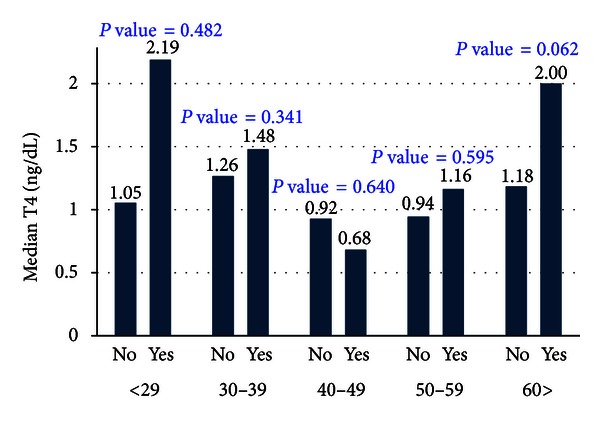
Thyroxine hormone (T_4_) levels among the immunological patients.

The thyroxine hormone (T_4_) levels were high for those subjects suffering from the autoimmune thyroid disease across all the age groups except for those aged 40–49 years as shown in Figure 3. 

**Figure 4:**
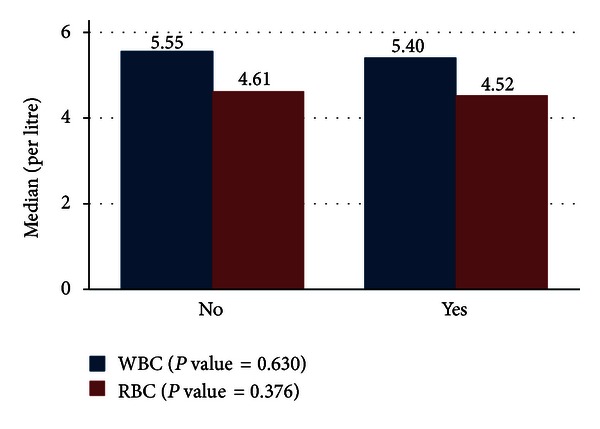
Levels of white blood cells and red blood cells for the immunological thyroid disease patients.

The white blood cells and the red blood cells levels in the group suffering from autoimmune thyroid disease were low, see Figure 4. 

**Figure 5:**
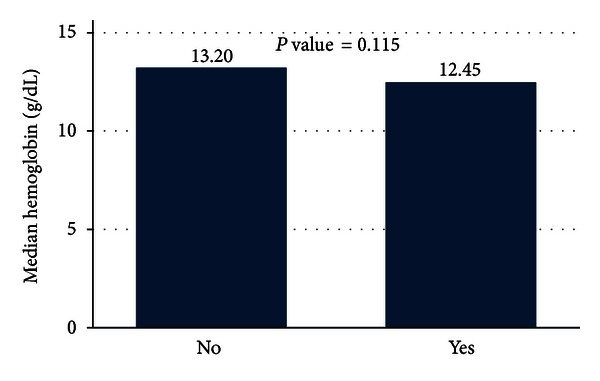
Levels of hemoglobin for the immunological thyroid disease patients.

The hemoglobin level of the subjects suffering from immunological thyroid disease was also low, Figure 5, and the differences were not significant for the two groups (*P*  value = 0.115).

**Figure 6:**
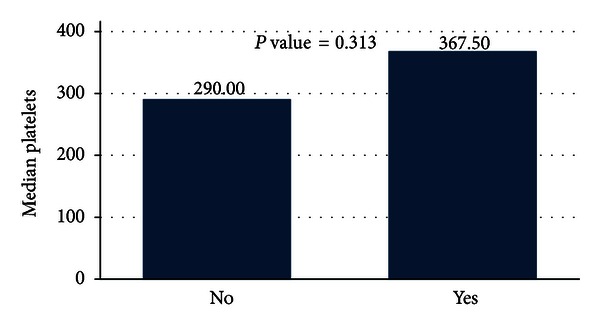
Levels of platelets for the immunological patients units.

The platelets levels were high for the subjects suffering from the immunological thyroid disease (Figure 6), but these differences were not statistically significant for the two groups (*P* value = 0.313).

## 4. Discussion

The analyses for this study included all patients aged at least 14 years who were undergoing thyroidectomy at Moi Teaching and Referral Hospital (MTRH), western Kenya, between 2008 and 2011. The primary outcome of interest was having autoimmune thyroid disease (thyroiditis). The independent variables were the demographic variables: age and sex; the thyroid hormones: TSH, T_3_, and T_4_; and hematological profiles.

We assessed the hematological profiles (WBCs, RBCs, HGB, and PLAT) in immunological thyroid disease patients. The white blood cells and the red blood cells levels in the group suffering from immunological thyroid disease were low. Likewise the hemoglobin level of the subjects suffering from immunological thyroid disease was low too. The platelets levels were high for the subjects suffering from the autoimmune thyroid disease. When the relationship between thyroid disorders and hematological profile was determined, the hematological profiles as well as the age for the immunological patients were not significantly different from those of the nonimmunological thyroid disease patients. This may be attributable to the small number of male patients in this study. Further the autoimmune thyroid disease is rare among the male population. Immunological conditions of those thyroid disorders are more common in females than in males. This explains the high number of females in this study. The disease is highly prevalent in females. Male cases are few. Apart from hormonal differences and cyclicity, the physiological roles of the thyroid gland are common in the two cases. This explains why we included both sexes and not females alone. The 5% of the males samples will not make a difference as seen from the analysis done.

As far as the white blood cells and red blood cells count reduction are concerned, this shows that the bone marrow is depressed and that thyroid hormones play an important role in the regulation of the human hematopoiesis in the bone marrow. This fact has also been shown to be evident in other studies [2]. With regard to white blood cells, triodothyronine (T_3_) hormone has been proven to be a prerequisite for normal B-cell production in the bone marrow through its regulation of pro-B-cell proliferation [8]. The hemoglobin level of the subjects suffering from immunological thyroid was low too. This clinically indicates a state of anemia. Other studies have also shown that hypothyroidism causes anemia or hyperproliferation of immature erythroid progenitors, and the anaemia is usually macrocytic hypochromic anaemia [3]. The increase in the production of platelet can be attributed to the imbalance in the hematopoiesis which is a compensatory mechanism [8]. These observations have confirmed the association between immunological thyroid disease and hematopoiesis and that hematological parameters are altered in this condition. Also it gives credence to the rejection of our null hypothesis which states that hematological profiles in immunological thyroid diseases patients do not differ from those without immunological thyroid disease.

## 5. Conclusion

The presence of anaemia in thyroid disease is associated with thyroid hormone. Therefore, thyroid hormones have a significant influence on erythropoiesis. The finding suggests that the molecular mechanism by which thyroid hormones influence hematopoiesis may provide a basis for therapeutic intervention in thyroid diseases. Immunological thyroid disease patients tend to have higher levels of thyroid hormonal profiles and low hematological profiles (WBCs, RBCs, and HB) though higher platelets compared to the nonimmunological thyroid disease patients.

## Figures and Tables

**Table 1:** Distribution of the demographic characteristics, hormones, and hematological properties among the immunological thyroid disease patients.

		Overall	Immunological status				Test for difference
Variable			Immunological		Nonimmunological	
	Sample size (*n*)	Median (Q1–Q3)	Sample size (*n*)	Median (Q1–Q3)	Sample size (*n*)	Median (Q1–Q3)	P values
Age	369	40 (33–50)	22	40 (32–54)	347	41 (32–50)	0.787
TSH	333	1.4 (0.7–2.5)	22	2.6 (109–3.6)	311	1.5 (0.7–2.6)	0.323
T_3_	319	1.67 (0.92–2.8)	21	2.6 (1.7–3.1)	298	1.4 (0.9–3.2)	**0.009**
T_4_	327	1.08 (0.7–1.7)	21	1.4 (1.0–2.0)	306	1.01 (0.7–1.8)	0.181
WBC	252	5.2 (4.1–7.0)	23	5.4 (4.2–8.5)	229	5.5 (4.4–7.5)	0.547
RBC	252	4.6 (4.4–4.8)	23	4.5 (4.4–4.7)	229	4.6 (4.4–4.8)	0.205
HB	252	13.1 (11.6–13.9)	23	12.5 (9.8–13.6)	259	13.2 (12.4–13.9)	0.291
Plat	260	292 (224–390)	22	367.5 (220.5–522.5)	238	290 (226.5–365.5)	0.488

**Table 2:** Relationship between thyroid disorder and hematological profiles among the male patients.

Variable	Sample size	Median (IQR)	Immunological (*n* = 9, 45%)	Nonimmunological (*n* = 11, 55%)	Wilcoxon rank-sum test (*P* value)
Age	19	52 (37–73)	49 (39–68)	62 (32–74)	0.788
TSH	17	1.2 (0.6–2.4)	1.6 (0.2–2.8)	1.1 (0.8–2.5)	0.474
T_3_	16	1.6 (0.5–3.5)	0.9 (0.1–3.8)	1.6 (1.3–3.3)	0.657
T_4_	16	0.6 (0.2–1.6)	0.7 (0.2–1.2)	0.5 (0.2–2.2)	0.614
Wbc	1	5.1 (4.5–6.2)	5.6 (4.8–6.1)	4.8 (4.4–6.2)	0.782
Rbc	11	4.8 (4.4–4.8)	4.6 (4.5–4.9)	4.8 (4.4–4.8)	0.853
Hb	13	13.4 (11.8–13.8)	13.1 (10.3–14.0)	13.4 (11.6–13.8)	0.826
Platelets	12	345 (264–365)	345 (278–365)	307 (262–415)	0.621
